# Pulmonary valve infective endocarditis with atrial septal defect and pulmonary valve disease—too coincidental to be true?

**DOI:** 10.1007/s12471-020-01369-2

**Published:** 2020-02-07

**Authors:** M. W. L. Smits, R. Tukkie, P. G. Meregalli, D. Robbers-Visser, P. T. G. Bot

**Affiliations:** 1 Department of Cardiology, Spaarne Gasthuis, Haarlem, The Netherlands; 2 Department of Cardiology, Amsterdam University Medical Centers—location AMC, Amsterdam, The Netherlands

**Fig. 1:**
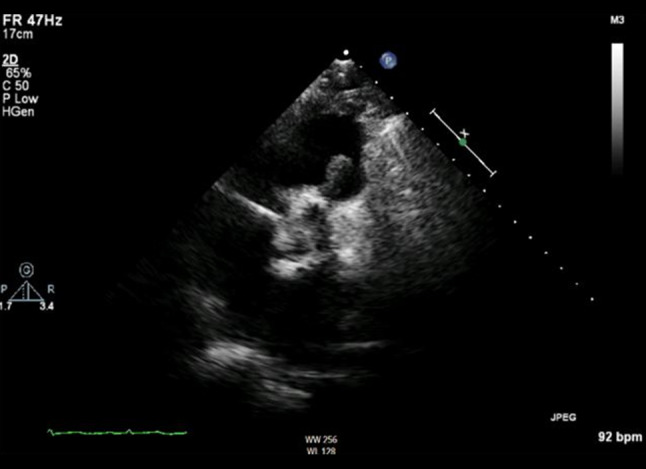
TTE showing a mobile structure on the pulmonary valve. *TT* transthoracic echocardiography

In this case we describe a 68-year-old male patient who presented with malaise after being treated for a urinary tract infection caused by *enterococcus faecalis*. The electrocardiogram showed atrial fibrillation and subsequent transthoracic echocardiography revealed a mobile structure on the pulmonary valve with an increased transpulmonary valve gradient (Fig. 1). Blood cultures were positive for *enterococcus faecalis *and PET-CT revealed F‑18-fluorodeoxyglucose (FDG) uptake in the right ventricular outflow tract and lungs.

**Fig. 2:**
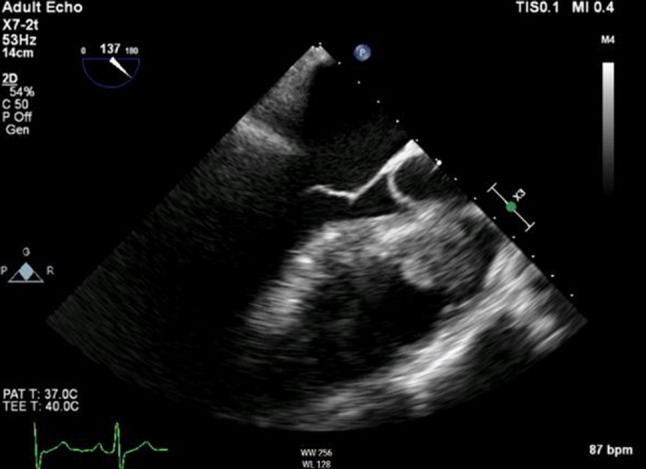
TEE showing thickened pulmonary valve and atrial septal defect. *TEE* transoesophageal echocardiography

Transoesophageal echocardiography revealed a previously unknown small atrial septal defect type 2 with left-to-right shunt as well as a moderate pulmonary valve stenosis and severe regurgitation (Fig. 2).

In the majority of cases, right-sided infective endocarditis involves the tricuspid valve and is associated with intravenous drug use or the presence of pacemakers [1–3]. In this case, a type 2 atrial septal defect and a dysplastic pulmonary valve were observed. This case underscores the importance of thorough investigation of coexistent congenital heart defects in cases of right-sided infective endocarditis [4].
